# Mental Rotation of Digitally-Rendered Haptic Objects

**DOI:** 10.3389/fnint.2019.00007

**Published:** 2019-03-14

**Authors:** Ruxandra I. Tivadar, Tom Rouillard, Cédrick Chappaz, Jean-François Knebel, Nora Turoman, Fatima Anaflous, Jean Roche, Pawel J. Matusz, Micah M. Murray

**Affiliations:** ^1^The Laboratory for Investigative Neurophysiology (LINE), Department of Radiology and Clinical Neurosciences, University Hospital Center and University of Lausanne, Lausanne, Switzerland; ^2^Department of Ophthalmology, Fondation Asile des Aveugles, Lausanne, Switzerland; ^3^Hap2u, Saint-Martin-d’Hères, France; ^4^Electroencephalography Brain Mapping Core, Center for Biomedical Imaging (CIBM) of Lausanne and Geneva, Lausanne, Switzerland; ^5^Information Systems Institute at the University of Applied Sciences Western Switzerland (HES-SO Valais), Sierre, Switzerland; ^6^Department of Hearing and Speech Sciences, Vanderbilt University, Nashville, TN, United States

**Keywords:** haptic, object, multisensory, mental rotation, sensory substitution, low vision, vision impairment

## Abstract

Sensory substitution is an effective means to rehabilitate many visual functions after visual impairment or blindness. Tactile information, for example, is particularly useful for functions such as reading, mental rotation, shape recognition, or exploration of space. Extant haptic technologies typically rely on real physical objects or pneumatically driven renderings and thus provide a limited library of stimuli to users. New developments in digital haptic technologies now make it possible to actively simulate an unprecedented range of tactile sensations. We provide a proof-of-concept for a new type of technology (hereafter haptic tablet) that renders haptic feedback by modulating the friction of a flat screen through ultrasonic vibrations of varying shapes to create the sensation of texture when the screen is actively explored. We reasoned that participants should be able to create mental representations of letters presented in normal and mirror-reversed haptic form without the use of any visual information and to manipulate such representations in a mental rotation task. Healthy sighted, blindfolded volunteers were trained to discriminate between two letters (either L and P, or F and G; counterbalanced across participants) on a haptic tablet. They then tactually explored all four letters in normal or mirror-reversed form at different rotations (0°, 90°, 180°, and 270°) and indicated letter form (i.e., normal or mirror-reversed) by pressing one of two mouse buttons. We observed the typical effect of rotation angle on object discrimination performance (i.e., greater deviation from 0° resulted in worse performance) for trained letters, consistent with mental rotation of these haptically-rendered objects. We likewise observed generally slower and less accurate performance with mirror-reversed compared to prototypically oriented stimuli. Our findings extend existing research in multisensory object recognition by indicating that a new technology simulating active haptic feedback can support the generation and spatial manipulation of mental representations of objects. Thus, such haptic tablets can offer a new avenue to mitigate visual impairments and train skills dependent on mental object-based representations and their spatial manipulation.

## Introduction

In everyday life, vision supports crucial functions that enable us to successfully interact with our environment, such as manipulation of objects as well as spatial orientation and navigation in space. These functions depend on the correct acquisition and maintenance of mental representations of our environment and the objects within it. In sighted individuals, vision typically predominates these functions and spatial abilities more generally (e.g., Welch and Warren, 1980; Knudsen and Knudsen, 1989; Schutz and Lipscomb, 2007). However, visual impairments affect nearly 300 million people globally, with another ~36 million suffering from complete loss of vision (World Health Organization, 2000). This calls for effective rehabilitation methods, including sensory substitution approaches.

Studies in visually impaired individuals document the extensive neuroplasticity of both non-visual functions, as well as within visual cortices. For example, visual deprivation enhances tactile acuity not only in sighted individuals (Pascual-Leone and Hamilton, 2001; Merabet et al., 2007; Norman and Bartholomew, 2011), but also in blind and visually impaired patients (Goldreich and Kanics, 2003; Lederman and Klatzky, 2009). It is now well-established that cross-modal plasticity can promote functions that are supported predominantly by vision. Tactile information has been most widely utilized to date to train functions such as reading (e.g., Braille) and exploration of space (e.g., white cane). Specifically, object geometry and form judgments based on haptics have been demonstrated to activate visual areas along the so-called dorsal pathway (Prather et al., 2004; Sathian, 2005). Furthermore, visual areas have been found to be activated during Braille reading in functional imaging studies (Sadato et al., 1996, 1998, 2002; Burton et al., 2002; Amedi et al., 2003). Sathian et al. (1997) were the first to demonstrate, *via* haemodynamic imaging, that discrimination of orientation of tactile gratings activates the same extrastriate areas as those observed active during visual orientation discrimination. This cross-modal functional recruitment of nominally visual cortices for tactile perceptual functions most likely results from cross-modal plasticity operating *via* the interplay between unisensory and multisensory neurons (Amedi et al., 2001; Kitada et al., 2006). More generally, there is now convergent and consistent evidence for visual cortex activation during tactile perception in both blind and sighted individuals (reviewed in Lacey and Sathian, 2014).

In addition to evidence pointing to the involvement of visual cortices in tactile discrimination, spatial functions can also be achieved in a modality-independent fashion, including based solely on tactile information. For example, studies of mental rotation where participants need to judge whether an image is portrayed in its normal or mirror-reversed form demonstrate a typical increase in reaction times (RTs) with increasing rotation of the image (Shepard and Metzler, 1971; Lacey et al., 2007a,b). Marmor and Zaback (1976) showed that the same mental rotation effect occurs with tactile stimuli. This and other findings have led to the belief that spatial properties can be encoded in a modality-independent format (Lacey and Campbell, 2006), and may thus engage a common spatial representational system (Lacey and Sathian, 2012; Lee Masson et al., 2016).

The discovery of modality-independence of spatial representations has opened a new avenue for vision rehabilitation, i.e., tactile-based sensory substitution. One particularly striking example here is the successful use of haptic stimulation of the tongue with the tongue-display unit (TDU) to retrain “tactile-visual” acuity (TDU, Chebat et al., 2007). The TDU is a sensory substitution device (SSD) that converts a visual stimulus into electro-tactile pulses delivered to the tongue *via* a grid of electrodes. Visually impaired individuals were able to discriminate various orientations of the letter E (i.e., the Snellen E test) based solely on stimulation with the TDU (Chebat et al., 2007). While such efforts are impressive, they risk remaining limited in their applications. However, this is at least partially addressed in new technologies for digitization of information, such as tablets digitally rendering tactile information (e.g., Xplore Touch^1^). This digitization of information has led to significant improvements in healthcare, including reduced costs and increased accessibility and reliability of treatments (Noffsinger and Chin, 2000; Dwivedi et al., 2002). Currently, visually impaired individuals require persistent training for the rehabilitation of visual functions that support basic everyday activities such as cooking, cleaning, and navigating one’s environment. This involves numerous hours of work together with therapists. Digitalizing the method of delivery of therapeutic procedures would likely allow visually impaired patients to be more independent and, so, successful, in their training. For one, the therapeutic programs could be created online and then easily downloaded onto a digital device. Second, patients would be able to practice and improve their tactile acuity as well as their form and object perception abilities without the constant presence of a therapist.

It is known that spatial operations such as mental rotation can be supported solely by tactile stimuli such as Plexiglas forms or wooden blocks (Marmor and Zaback, 1976; Carpenter and Eisenberg, 1978, for recent reviews see Prather and Sathian, 2002; Lacey et al., 2007a). By contrast, it is unknown whether individuals can create and manipulate mental representations of objects based solely on *simulated* haptic representations. If spatial functions can be rehabilitated with digital devices, this should substantially improve both the speed and the extent of the recovery as well as the independence of visually impaired patients. Haptic tablets thus promise to open up unprecedented possibilities for recovery of visual functions for blind and visually impaired individuals, due to the ease of delivery of digital information and of the transfer of the learnt information from tablet to veridical environments. Being able to mentally rotate digitally presented haptic objects would serve as an important proof-of-concept for the successful acquisition of a representation of a simulated haptic space.

To this end, the present study investigated whether participants would be able to successfully mentally rotate representations of letters in their normal and mirror-reversed forms, experienced solely *via* digitally-rendered haptic feedback. We focused on the distinction of letter forms (i.e., normal vs. mirror-reversed), because judgments of letter identity (for example the distinction between a letter and a number) do not necessarily implicate mental rotation (White, 1980). We hypothesized that normally-sighted participants should show the prototypical mental rotation effect, with steadily decreasing accuracy (and increasing RTs) with increasing angular disparity from the prototypical upright letter orientation, which would translate into a main effect of angle. Moreover, we expected that participants would show better performance with letters in their normal form compared to mirror-reversed letters, due to the well-investigated effect of stimulus familiarity on mental rotation (White, 1980; Bethell-Fox and Shepard, 1988; Prather and Sathian, 2002). We also expected a main effect of training, meaning that participants would perform better with letters which they had trained with, compared to letters that were untrained.

## Materials and Methods

### Participants

All participants provided written informed consent to procedures approved by the cantonal ethics committee in accordance with the Declaration of Helsinki. We tested 17 adults (12 women and five men; age range 25–37 years, mean ± stdev: 28.9 ± 3.5 years), who volunteered for our experiment. Participants reported normal or corrected-to-normal vision. No participant had a history of or current neurological or psychiatric illnesses. Handedness was assessed *via* the Short Form of the Edinburgh Handedness Inventory (Oldfield, 1971). Two of our participants were left-handed, while the remainder were right-handed. We also asked our participants about their experience playing a musical instrument, due to evidence of increased cortical representation of the hands of musical instrument players (see e.g., Elbert et al., 1995). Nine participants were active instrument players (i.e., actively played instruments at the time of the testing session), five had formerly played instruments (i.e., during childhood, adolescence and early adulthood, however they were not actively practising at the time of testing), and three played no instruments.

### Apparatus

Haptic stimulation was delivered *via* a tablet with a TFT capacitive 7-inch touchscreen with a resolution of 1,024 × 600 pixels. The screen of the tablet is controlled by a Raspberry Pi 3 based system, and the operating system is Raspbian (Linux). The processor of the tablet is a Broadcom ARMv7, quadcore 1.2 GHz and it has 1 Go RAM and Rev C WaveShare. The tablet comes with a haptic creation tool, which is a software that allows for user control of haptic textures. Several other APIs based on C++ or Java are installed, such as library tools that allow the implementation of haptics on other applications. Figures in jpeg format were re-coded in haptic format using a kit written in C++. For more technical details describing the rendering of the haptic feedback, see Vezzoli et al. (2016, 2017) and Rekik et al. (2017).

### Stimuli

**Figure 1:**
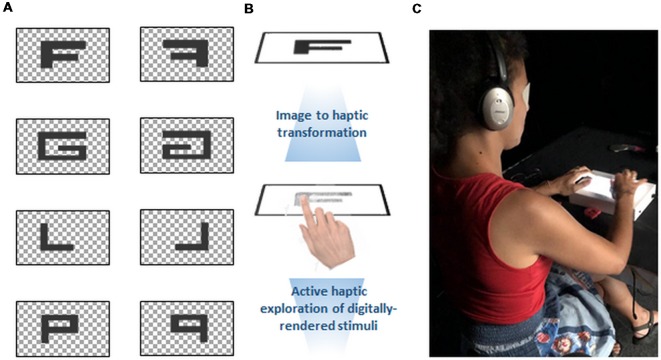
**(A)** Stimuli used in the experiment. These images are based on reverse translation of the haptic “image.” The checkered portions refer to regions with no haptic texture. The letters were created to have the same proportions on the haptic tablet screen, and thus they appear slightly distorted. Normal stimuli and their mirror images were rotated at 90°, 180°, 270° and were individually presented to participants on the tablet. **(B)** Transformation of the stimuli into haptic renderings was possible *via* a pre-installed kit. The transformation takes a cell (8 × 8 pixels) from the picture file and codes the cells into textures with the help of a haptic library where different textures are defined. Participants were then able to feel the vibrations on the tablet screen only at those places where the cells were transformed. **(C)** Experimental setup. Participants had their eyes blindfolded and wore noise-canceling headphones in order to prevent any other external stimulation interfering with the haptic sensation. After exploring the letter on the tablet for 30 s with a single finger, they indicated if the letter was normal or mirrored *via* a computer mouse button-press with their non-dominant hand, which would then initiate the passage to the next trial.

Stimuli consisted of four capital letters—L, P, F and G—created in Paint (see e.g., Carpenter and Eisenberg, 1978; see also Figure 1). We chose these capital letters as their mirror-image counterparts do not confuse, as compared to for example lower-case “d,” whose mirror image is “b” and “b,” whose mirror image is “d” (Corballis and McLaren, 1984). Moreover, these letters have previously been used in mental rotation tasks (Cohen and Polich, 1989; Rusiak et al., 2007; Weiss et al., 2009), including tasks with tactile objects (e.g., Carpenter and Eisenberg, 1978). The letters were resized to always be presented centrally on the screen of the haptic tablet, which has a pixel resolution smaller than that used to generate the images. Letters were then rotated to 0°, 90°, 180° and 270° and mirrored in Matlab. Letter size was 935 × 509 pixels. With regard to the image-to-haptic conversion, the letters appeared centrally on a white background. White pixels did not produce the feeling of a texture on a finger (i.e. “empty” pixels). All non-white pixels were then coded with the same haptic texture, which was created using the hap2u pre-installed Texture Editor software. The ultrasonic vibration was adjusted to have a square shape, as this offers the most intense and quick reduction of the friction of the screen under the finger, thus conferring a rather sharp and pointy sensation, in contrast to a sinusoidal-shaped wave, which would confer a rather smooth perception. The period of the window of one square ultrasonic signal was chosen to be 3,500 μm (which is considered a “coarse” texture, see Hollins and Risner, 2000), and the amplitude was set at 100%, meaning roughly 2 μm (as the friction reduction hits a plateau at this value, see e.g., Sednaoui et al., 2017).

### Procedure and Task

Participants were tested in a sound-attenuated, darkened room (WhisperRoom MDL 102126E). Subjects were blindfolded and wore noise-canceling headphones (Bose model QuietComfort 2), in order to block any residual light and the sounds of the ultrasonic vibrations produced by the tablet. None of the participants had any prior visual or haptic exposure to the stimuli used in the paradigm, minimizing any cross-modal facilitation (Lacey et al., 2007a,b). The participant’s task was a two-alternative forced choice that required discrimination of normal and mirror-reversed letters *via* a mouse click (left mouse press for the normal form, right mouse press for the mirrored form; same for all participants). Participants were instructed to use a finger from their dominant hand for tablet exploration, and the non-dominant one for responses. The task was to feel the letter on the haptic tablet for 30 s, recognize the letter, and if needed, to mentally rotate the letter to the 0° form, in order to decide whether the normal or the mirror-reversed form had been presented. We used explicit instructions, since it has been reported that this is not a determinant of whether a mental rotation effect is observed (reviewed in Prather and Sathian, 2002). Stimuli were presented for a duration of 30 s. Next, participants had 20 s for responding, and were instructed to respond as quickly and as accurately as possible. After the response, the next trial was initiated and was preceded by an inter-trial interval randomly ranging between 500 and 1,000 ms. Each participant completed three blocks of training, each comprising 16 trials (two per condition; informed by a pilot study). Participants were trained on pairs of two letters—either L and P or F and G—that they were assigned in a counterbalanced manner across individuals. We grouped these letters given their perceptual closeness, which allowed a progressive learning procedure. We decided to focus the training on a particular letter pairing in order to investigate skill transfer to new, untrained stimuli. Participants were first trained to explore the tablet screen *via* lateral sweeps [(Stilla and Sathian, 2008), see e.g., (Lederman and Klatzky, 1993) for a discussion of which tactile exploration strategies are particularly appropriate to disclose specific object characteristics, and (Hollins and Risner, 2000) for a discussion of how dynamic vs. static exploration affects coarse (>100 μm) as compared to fine texture discrimination], using only one finger at a time. Subjects were allowed to change the finger they used for exploration, due to a common complaint about adaptation of their tactile sensation during the pilot experiments or during the training blocks. However, they were not allowed to change the hand used for exploration. Subjects were then taught how to discriminate horizontal from vertical lines, and finally, how to discriminate between the two letters that they were trained on. The experimenter gave subjects verbal instructions and feedback throughout the training session. The testing phase comprised four blocks of 32 trials, making 128 trials in total per participant (i.e., eight trials per each condition, in total 16 conditions). During the experiment, participants were allowed to take regular breaks between blocks of trials to maintain high concentration and prevent fatigue. Stimulus delivery and behavioral response collection were controlled by Psychopy software (Peirce, 2007).

### Behavioral Analysis

Data were pre-processed in Matlab and analyzed in R (R Core Team, 2018) and SPSS (IBM Corp, 2017). First, we excluded all trials with RTs longer than 15 s (5% of trials), as well as missed trials (2.5% of trials), which were trials where a response was not given within 20 s. We then excluded any remaining outlier trials on a single subject basis (i.e., for each subject and condition), applying a mean ± 2 standard deviations criterion to their RTs (2.7% of trials, see Ratcliff, 1993; Field et al., 2012). Accuracy was then calculated. RT data were not further analyzed, since responses were only provided after stimulus offset followed by a subsequent cue. Data from three participants were excluded due to very low accuracy for the 0° condition (<50%). We compared Accuracy with a 2 × 2 × 4 repeated measures ANOVA with factors Training (trained/untrained), Condition (normal/mirror) and Angle (0°, 90°, 180°, 270°), after not having found a significant deviation from the Normal distribution and from homoscedasticity.

## Results

**Figure 2:**
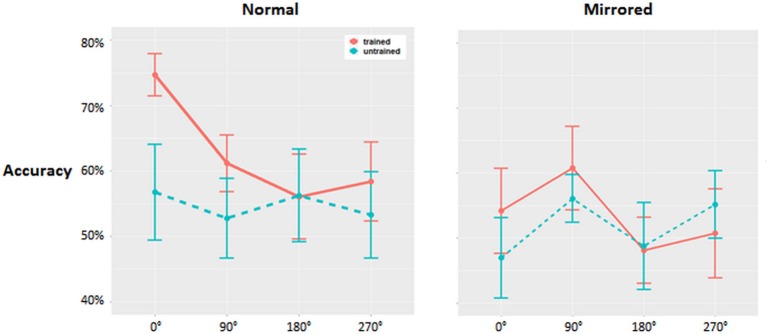
Group-averaged (*N* = 14) accuracy data for normal and mirrored stimuli (SEM indicated). The left column displays results for normal stimuli, while the right displays results for mirrored stimuli. Red lines refer to trained stimuli, while the blue lines represent untrained stimuli.

Mean accuracy rates are displayed in Figure 2. The 2 × 2 × 4 ANOVA with factors of Training (trained/untrained), Condition (normal/mirror) and Angle (0°, 90°, 180°, 270°) revealed a significant interaction and two main effects. The Angle × Trained interaction was significant (*F*_(1,13)_ = 4.912; *p* < 0.05, $\eta_{\text{p}}^{2}$ = 0.274), and there were main effects of Training (*F*_(1,13)_ = 5.88; *p* = 0.03, $\eta_{\text{p}}^{2}$ = 0.314), with generally higher accuracy scores for trained vs. untrained letters, and Condition (*F*_(1,13)_ = 6.02; *p* = 0.02, $\eta_{\text{p}}^{2}$ = 0.317), with generally higher accuracy scores for normal compared to mirrored stimuli. Given this significant interaction, we carried out separate 2 × 4 ANOVAs (Condition × Angle) for trained and untrained letters. Untrained letters revealed no interactions or main effects (*F* ≤ 0.6). By contrast, trained letters exhibited a main effect of Condition (*F*_(1,13)_ = 11.46, *p* < 0.01, $\eta_{\text{p}}^{2}$ = 0.470) and a main effect of Angle (*F*_(3,13)_ = 6.625, *p* = 0.02, $\eta_{\text{p}}^{2}$ = 0.338). Trained letters in their normal form had higher accuracy scores compared to trained letters in their mirrored form, and accuracy generally decreased with increasing angular disparity. Performance on untrained normal letters was more similar to performance on mirrored letters than to normal trained letters.

## Discussion

We provide the first demonstration that digitally-rendered haptic stimuli can support the creation of mental representations of objects that can then be spatially manipulated. Participants’ accuracy scores decreased with greater angular disparity of the presented letters from upright, indicating a prototypical mental rotation effect for trained letters (Shepard and Metzler, 1971). Moreover, letters in their mirrored form were less accurately detected compared to letters in their normal form, consistent with the stimulus familiarity effect that has been previously found to influence mental rotation with real visual stimuli (White, 1980). Specifically, normally sighted participants performed significantly better when tested on previously trained compared to untrained letters. This effect was observed for letters presented in their canonical form, and less for letters in their mirrored form. In addition, our results show that a short training session of about 45 min on the haptic tablet was sufficient to significantly increase the ability to correctly identify the correct form of haptic letters. These results extend previous efforts to support rehabilitation of spatial functions using SSDs, and open new avenues for applications of digital haptic technology.

Mental rotation of objects created by haptic feedback successfully modulated accuracy of object recognition; increasing angular disparity away from the prototypical orientation linearly reduced recognition accuracy. As expected, performance was significantly higher for normal letters, compared to mirrored letters, and for trained letters, as compared to untrained letters. Accuracy for letters in their normal upright form decreased up to 180°, with a slight increase for stimuli rotated at 270°. Similar results have previously been found in mental rotation tasks with stimuli of different kinds (see e.g., Kosslyn et al., 1998; Hyun and Luck, 2007; Milivojevic et al., 2011; Zeugin et al., 2017), further corroborating that our experimental manipulation was effective and that mental rotation of our haptic letter stimuli indeed took place. The significant interaction between factors Condition and Angle illustrates the fact that mental rotation of familiar stimuli was more successful than for unfamiliar stimuli. To be precise, given that the stimuli were letters, they can generally be considered familiar stimuli, however only letters presented in their normal form can be considered overlearned (White, 1980), while letters in their mirrored form can be considered unfamiliar, as individuals are seldomly using mirrored letters in their everyday lives. In addition, the significant effect of the factor Training indicates that with only little training on the task and limited exposure to haptic stimulation before the testing, participants were able to improve their performance, which was not the case for untrained letters.

Our findings replicate and extend prior studies of mental rotation based on haptic information. Mental rotation has been studied with Plexiglas letters and objects (Carpenter and Eisenberg, 1978; Hunt et al., 1989), abstract Braille-like stimuli (Röder et al., 1997), as well as with haptic versions of the Shepard and Metzler (1971) stimuli (Robert and Chevrier, 2003). These and other similar works have likewise shown that performance worsens with increasing angular displacement from upright, independently of whether an explicit instruction was provided to use a strategy based on mental rotation (reviewed in Prather and Sathian, 2002). By contrast, evidence of mental rotation with tactile stimuli does appear to vary with task. Tasks requiring mirror-image discrimination yield mental rotation effects, whereas those requiring identification of isolated stimuli generally do not (Prather and Sathian, 2002). Our study required participants to discriminate whether each stimulus was normal vs. mirror-reversed, and we indeed observed a mental rotation effect for trained letters. Our accuracy rates are consistent with, albeit somewhat lower than, what has been reported in sighted participants presented with physical objects (~80%–90% in Marmor and Zaback, 1976; Röder et al., 1997; Robert and Chevrier, 2003). However, two important distinctions in our study are the use of digital haptics, and moreover, that participants could only use a single finger to explore the stimulus. Ongoing efforts are working to enhance the haptic perceptual qualia as well as to permit exploration by multiple fingers simultaneously. Such notwithstanding, this limitation may nonetheless help us hone in on specific exploration and haptic learning strategies. Minimally, our results demonstrate that mental representations of haptic objects and their discrimination can be ascertained using information acquired with a single digit.

To summarize, our results indicate that participants were able to mentally manipulate internal representations of familiar stimuli that they learned solely in a haptic manner, through interaction with a digitally created texture. While our results have potential applications in the simulation of tactile sensorial perceptions in virtual reality, we do not have the space to discuss these at length here. Instead, we would like to focus on the important implications that our results have for cognitive models of spatial functions, as well as on the implications for the rehabilitation thereof in patients suffering from impairments due to vision loss. In what follows, we will discuss these latter two points.

### Implications for Models of Spatial Functions

Our results have implications for current models of cortical mechanisms that decode spatial characteristics of objects. Recently, evidence has been accumulating for a decoding mechanism that is modality-independent, with spatial features of objects and spaces being communicated through visual (Koenderink et al., 1992; Erens et al., 1993), haptic (Kappers and Koenderink, 1999; Prather et al., 2004; Snow et al., 2014; Lee Masson et al., 2018), and auditory (Amedi et al., 2007, 2002) information alone, as well as through multisensory information (Lacey et al., 2009; Sathian et al., 2011; Lacey and Sathian, 2014; Lee Masson et al., 2016, 2017). Moreover, it was demonstrated that multisensory regions, such as V1, IPS, and LOC, specifically encode spatial characteristics such as shape, but not object identity (Amedi et al., 2002). Our results further support such modality-independent models of spatial representations. In particular, it was possible for us to convey the shapes of haptic objects (i.e., letters) to participants through unisensory haptic stimuli. This indicates that spatial features of objects, and, specifically, of object shape, can be decoded from a variety of stimulus formats—be it visual, auditory, or somatosensory. However, sensory impressions coming from haptic and visual information are very different (Rose, 1994), and vision and touch use different metrics and geometries (Kappers and Koenderink, 1999). Nevertheless, there is substantial neuroimaging evidence showing that vision and touch are intimately connected even if there is no direct, one-to-one mapping (see Amedi et al., 2005; Sathian, 2005 for reviews). For one, cerebral cortical areas previously regarded as exclusively unisensory in nature are activated by sensory inputs in a task- and stimulus-specific manner (Lacey et al., 2007a). New evidence also supports high similarities between visual and haptic representations of object perceptual spaces (Cooke et al., 2007; Wallraven et al., 2014; Lee Masson et al., 2016). These results have been further complemented by neuroimaging studies, that helped in corroborating the result of high correlations between perceptual spaces reconstructed using tactile vs. visual information (Snow et al., 2014; Smith and Goodale, 2015). Indeed, clinical cortical lesion studies demonstrate that lesions of visual brain areas, such as the inferior occipito-temporal cortex, or the anterior intraparietal sulcus, are accompanied by tactile agnosia for objects, despite intact somatosensory cortical areas (Feinberg et al., 1986; James et al., 2002). Collectively, our results support a task-specificity, as compared to a stimulus-specificity, of spatial functions.

### Implications for Rehabilitation of Spatial Functions

Our study further validates efforts of rehabilitation of spatial functions through SSDs. Cross-modal and multisensory integration are the drivers of neuroplasticity in visual areas (Kirkwood et al., 1996; Amedi et al., 2004; Merabet et al., 2005; Pascual-Leone et al., 2005; Murray et al., 2015), which promotes a task-selective and modality-independent re-specialization of these cortical structures. Besides the known applications of tactile sensory substitutions such as the Braille alphabet, white cane, or the TDU, our results open new avenues for mitigation of deficiencies of spatial functions in the blind and visually impaired. Indeed, it has been demonstrated numerous times that tactile information can support spatial functions in blind, visually impaired, and sighted subjects (Marmor and Zaback, 1976; Carpenter and Eisenberg, 1978; Grant et al., 2000; Ptito et al., 2005; Sathian, 2005; Chebat et al., 2007; Wan et al., 2010; Rovira et al., 2011; Vinter et al., 2012). However, the main innovation introduced by our study is the digital simulated nature of the tactile stimuli. As digital information is easily recoded and reproduced, our results open new exciting venues for increased accessibility of traditionally visual functions, such as reading, navigation, etc., to visually impaired people.

In addition, such tactile substitution and multisensory techniques can also be used to retrain spatial functions after sight restoration. Specifically, patients with long-lasting cataracts have deficient depth perception after cataract removal (Hartung, 1962; Gregory, 2003; McKyton et al., 2015), despite normal low-level visual perception. Thus, as auditory information is unable to confer spatial information (Amedi et al., 2002), one could imagine complementing rehabilitation programs with tactile spatial information, in order to confer distance relations in a multisensory manner. Another exciting endeavor for further research that we are now also pursuing in the laboratory is the extent to which simulated haptic information can support the encoding of entire familiar and new spaces. In short, simulated tactile information has critical implications for applications in rehabilitation regimes. Besides being specifically able to convey spatial relations, as opposed to auditory information, simulated tactile stimuli have the added value of accessibility. This benefit renders tactile tablets a promising solution for the mitigation of complete or partial loss of spatial abilities due to sensory loss or deprivation.

## Conclusion

We trained normally-sighted participants on a haptic mirror-image discrimination task, using a new technology that digitally simulates texture. After only a short exposure and habituation to the new sensation, and relatively little training on the task, participants were able to mentally manipulate internal representations of the trained letters. This indicates that spatial functions and attributes such as object shape rely on a modality-independent mechanism, and that multiple sensory modalities are capable of supporting spatial computations. Furthermore, our results have important implications for research on virtual simulated sensorial perception, as well as for neural plasticity and visual rehabilitation, and highlight the merit of restoring visual functions through SSDs.

## Data Availability

The datasets generated for this study are available on request to the corresponding author.

## Author Contributions

RT and MM designed the study. TR and CC provided essential equipment. RT, MM, and J-FK set up the stimuli and paradigm. RT, MM and NT completed the data analysis. RT, FA, JR, and MM interpreted the results. All authors contributed to the writing and final revisions to the manuscript.

## Conflict of Interest Statement

CC is the CEO and Founder of Hap2U. CC thus has commercial, proprietary and financial interest in Hap2U, which provided the haptic tablet device instrument related to this article. TR is a paid employee of Hap2U. The remaining authors declare that the research was conducted in the absence of any commercial or financial relationships that could be construed as a potential conflict of interest.

## Footnotes

^1^http://www.hap2u.net
